# 2-Oxochromen-4-yl 4-(dimethyl­amino)­benzoate

**DOI:** 10.1107/S1600536811030844

**Published:** 2011-08-06

**Authors:** Akoun Abou, Abdoulaye Djandé, Bintou Sessouma, Adama Saba, Rita Kakou-Yao

**Affiliations:** a Laboratoire de Cristallographie et Physique Moléculaire, UFR SSMT, Université de Cocody 22 BP 582 Abidjan 22, Côte d’Ivoire; b Laboratoire de Chimie Bio-organique et Phytochimie, Université de Ouagadougou 03 BP 7021 Ouagadougou 03, Burkina Faso

## Abstract

In the title mol­ecule, C_18_H_15_NO_4_, the benzoate ring is oriented at a dihedral angle of 43.43 (6)° with respect to the planar [maximum deviation = 0.038 (2) Å] chromene ring. The crystal structure features *R*_2_^2^(12) centrosymetric dimers formed *via* C—H⋯O inter­actions and these dimeric aggregates are connected by C—H⋯π inter­actions.

## Related literature

For the biological activity of coumarin derivatives, see: Ukhov *et al.* (2001 ▶); Abd Elhafez *et al.* (2003 ▶); Basanagouda *et al.* (2009 ▶); Liu *et al.* (2008 ▶); Trapkov *et al.* (1996 ▶); Vukovic *et al.* (2010 ▶); Emmanuel-Giota *et al.* (2001 ▶); Hamdi & Dixneuf (2007 ▶); Wang *et al.* (2001 ▶); Marchenko *et al.* (2006 ▶). For hydrogen-bond graph-set motifs, see: Bernstein *et al.* (1995 ▶).
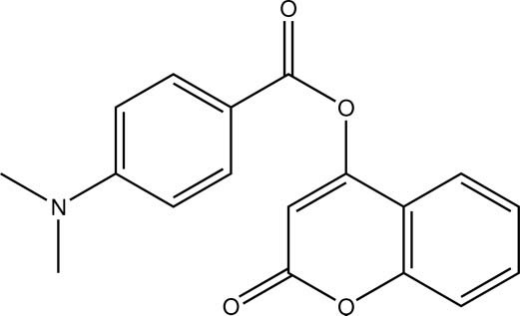


## Experimental

### 

#### Crystal data

C_18_H_15_NO_4_
*M* *_r_* = 309.32
Triclinic, 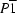

*a* = 7.4939 (2) Å
*b* = 10.2361 (3) Å
*c* = 10.6620 (3) Å
α = 92.307 (3)°
β = 103.935 (1)°
γ = 109.852 (2)°
*V* = 739.92 (4) Å^3^
*Z* = 2
Mo *K*α radiation
μ = 0.10 mm^−1^
*T* = 298 K
0.50 × 0.40 × 0.30 mm

#### Data collection

Nonius KappaCCD diffractometer
8424 measured reflections
3590 independent reflections
2897 reflections with *I* > 2σ(*I*)
*R*_int_ = 0.024

#### Refinement

*R*[*F* ^2^ > 2σ(*F* ^2^)] = 0.048
*wR*(*F* ^2^) = 0.120
*S* = 0.98
3585 reflections
208 parameters
H-atom parameters constrained
Δρ_max_ = 0.23 e Å^−3^
Δρ_min_ = −0.20 e Å^−3^

### 

Data collection: *COLLECT* (Nonius, 2001 ▶); cell refinement: *DENZO*/*SCALEPACK* (Otwinowski & Minor, 1997 ▶); data reduction: *DENZO*/*SCALEPACK*; program(s) used to solve structure: *SIR92* (Altomare *et al.*, 1994 ▶); program(s) used to refine structure: *CRYSTALS* (Betteridge *et al.*, 2003 ▶); molecular graphics: *PLATON* (Spek, 2009 ▶); software used to prepare material for publication: *CRYSTALS*.

## Supplementary Material

Crystal structure: contains datablock(s) global, I. DOI: 10.1107/S1600536811030844/tk2767sup1.cif

Structure factors: contains datablock(s) I. DOI: 10.1107/S1600536811030844/tk2767Isup2.hkl

Supplementary material file. DOI: 10.1107/S1600536811030844/tk2767Isup3.cml

Additional supplementary materials:  crystallographic information; 3D view; checkCIF report

## Figures and Tables

**Table 1:** Hydrogen-bond geometry (Å, °) *Cg*3 is the centroid of the C15–C18/C22/C23 ring.

*D*—H⋯*A*	*D*—H	H⋯*A*	*D*⋯*A*	*D*—H⋯*A*
C9—H91⋯O8^i^	0.96	2.49	3.449 (2)	171
C7—H71⋯*Cg*3^ii^	0.95	2.84	3.429 (2)	121
C20—H202⋯*Cg*3^iii^	0.99	2.91	3.777 (2)	146

Symmetry codes: (i) 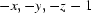; (ii) 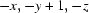; (iii) 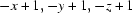.

**Fig. 1.**
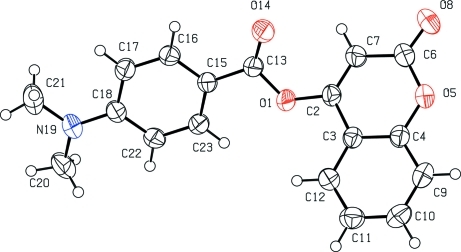
The molecular structure of (I) showing the atomic labeling scheme, with displacement ellipsoids drawn at the 50% probability level. H atoms are shown as spheres of arbitrary radius.

**Fig. 2.**
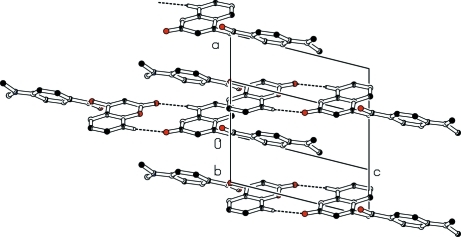
Crystal packing, viewed in projection down the b axis, showing parallel centrosymmetric dimers. Dashed lines indicate hydrogen bonds. H atoms not involved in hydrogen bonds have been omitted for clarity.

**Fig. 3.**
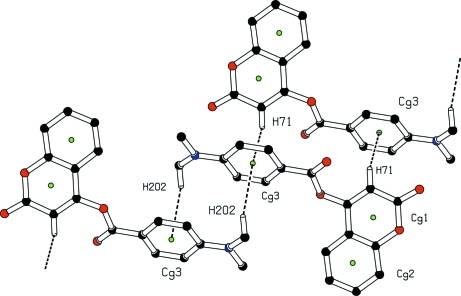
Crystal packing, showing C—H···π stacking interactions. The green dots are centroids of rings. H atoms not involved in C—H···π interactions have been omitted for clarity.

## supplementary crystallographic information

### Comment

Coumarin constitutes one of the major classes of naturally occurring compounds, and interest in its chemistry continues unabated because of its usefulness as biologically active agents. It also represents the core structure of several molecules of pharmaceutical importance. Coumarin and its derivatives have been reported to serve as anti-bacterial (Ukhov *et al.*, 2001; Abd Elhafez *et al.*, 2003; Basanagouda *et al.*, 2009; Liu *et al.*, 2008), anti-oxidant (Trapkov *et al.*, 1996; Vukovic *et al.*, 2010), anti-inflammatory (Emmanuel-Giota *et al.*, 2001; Hamdi & Dixneuf, 2007), anti-coagulant (Hamdi *et al.*, 2007) and anti-tumour (Wang *et al.*, 2001; Marchenko *et al.*, 2006) agents. Therefore, the synthesis of new coumarin derivatives is of considerable interest. In order to study the influence of new substituents on the activity of the coumarin derivative, the title compound, (I), has been synthesized and in this paper, we present its molecular structure, Fig. 1.

In (I), the planar chromene ring system resulting from the two coupled rings (benzene and 3,6-dihydro-2*H*-pyran) is oriented with respect to the benzoate-benzene ring at a dihedral angle of 43.43 (6)°. Atoms O14, N19, C13 and C21 are 0.046 (1), 0.052 (1), 0.079 (2) and 0.077 (3) Å out of the plane of the benzoate-benzene ring, respectively, so, they are coplanar with this ring.

In the crystal structure, intermolecular C—H···O interactions (Table 1) link the molecules into centrosymmetric dimers through *R*_2_^2^(12) ring motifs (Bernstein *et al.*, 1995) (Fig. 2). Two weak C—H···π interactions formed between the H71 and H202 atoms and the centroid *Cg*3 of the benzoate-benzene ring (Table 1 and Fig. 3) further stabilize the structure.

### Experimental

To a solution of 4.10^-2^ mole of paradimethylamino benzoyl chloride in 150 ml of dried tetrahydrofuran, was added 0.12 mole of dried triethylamine and 4.10^-2^ mole of 4-hydroxycoumarin by small portions over 30 min. The mixture was then refluxed for 3 h and poured in 300 ml of chloroform or dichloromethane. The solution was acidified with dilute hydrochloric acid until the pH was 2 or 3. The organic layer was extracted, washed with water, dried over MgSO_4_ and the solvent removed. The crude product was recrystallized in chloroform. Colourless crystals of the title compound are obtained in a good yield: 82.6%; M.pt. 445 K.

### Refinement

The H-atoms were placed at calculated positions and were included in the refinement in the riding model approximation with C—H in the range of 0.94–0.99 Å, and with *U*_iso_(H) = 1.2–1.5*U*_eq_(C).

### Crystal data

**Table:**

C_18_H_15_NO_4_	*Z* = 2
*M**_r_* = 309.32	*F*(000) = 324
Triclinic, *P*1	*D*_x_ = 1.388 Mg m^−^^3^
Hall symbol: -P 1	Melting point: 445 K
*a* = 7.4939 (2) Å	Mo *K*α radiation, λ = 0.71073 Å
*b* = 10.2361 (3) Å	Cell parameters from 8424 reflections
*c* = 10.6620 (3) Å	θ = 2.0–28.7°
α = 92.307 (3)°	µ = 0.10 mm^−^^1^
β = 103.935 (1)°	*T* = 298 K
γ = 109.852 (2)°	Parallelepiped, colourless
*V* = 739.92 (4) Å^3^	0.50 × 0.40 × 0.30 mm

### Data collection

**Table:**

Nonius KappaCCD diffractometer	2897 reflections with *I* > 2σ(*I*)
Radiation source: fine-focus sealed tube	*R*_int_ = 0.024
graphite	θ_max_ = 28.7°, θ_min_ = 2.0°
φ and ω scans	*h* = −9→9
8424 measured reflections	*k* = −13→13
3590 independent reflections	*l* = −14→14

### Refinement

**Table:**

Refinement on *F*^2^	Primary atom site location: structure-invariant direct methods
Least-squares matrix: full	Secondary atom site location: difference Fourier map
*R*[*F*^2^ > 2σ(*F*^2^)] = 0.048	Hydrogen site location: inferred from neighbouring sites
*wR*(*F*^2^) = 0.120	H-atom parameters constrained
*S* = 0.98	Method = Modified Sheldrick *w* = 1/[σ^2^(*F*^2^) + (0.05*P*)^2^ + 0.22*P*], where *P* = [max(*F*_o_^2^,0) + 2*F*_c_^2^]/3
3585 reflections	(Δ/σ)_max_ = 0.00023
208 parameters	Δρ_max_ = 0.23 e Å^−^^3^
0 restraints	Δρ_min_ = −0.20 e Å^−^^3^
60 constraints	

### Special details

**Table:**

Refinement. The 5 reflections 1 0 0; 0 1 0; -1 0 1; 0 0 1; -1 1 1 have been measured with too low intensities. It might be caused by some systematical error, probably by shielding by a beam stop of these diffractions. They were not used in the refinement.

### Fractional atomic coordinates and isotropic or equivalent isotropic displacement parameters (Å^2^)

**Table:**

	*x*	*y*	*z*	*U*_iso_*/*U*_eq_	
O1	0.16941 (15)	0.34698 (9)	−0.00111 (8)	0.0462	
C2	0.13553 (19)	0.27238 (13)	−0.11979 (11)	0.0385	
C3	0.16678 (17)	0.14123 (12)	−0.10953 (12)	0.0363	
C4	0.12326 (18)	0.05485 (13)	−0.22545 (12)	0.0396	
O5	0.05704 (15)	0.09384 (10)	−0.34457 (9)	0.0493	
C6	0.0349 (2)	0.22168 (15)	−0.35487 (13)	0.0480	
C7	0.0715 (2)	0.31027 (14)	−0.23575 (13)	0.0453	
O8	−0.0149 (2)	0.24941 (13)	−0.46358 (10)	0.0703	
C9	0.1466 (2)	−0.07411 (14)	−0.22593 (15)	0.0499	
C10	0.2154 (2)	−0.11591 (15)	−0.10867 (16)	0.0531	
C11	0.2611 (2)	−0.03116 (15)	0.00810 (15)	0.0504	
C12	0.2373 (2)	0.09635 (14)	0.00783 (13)	0.0436	
C13	0.26101 (19)	0.49181 (13)	0.02252 (12)	0.0394	
O14	0.31022 (19)	0.56038 (11)	−0.06000 (10)	0.0605	
C15	0.29273 (18)	0.54219 (12)	0.15893 (11)	0.0361	
C16	0.40333 (19)	0.68335 (13)	0.20353 (12)	0.0399	
C17	0.4486 (2)	0.73643 (14)	0.33261 (13)	0.0438	
C18	0.38190 (19)	0.64988 (14)	0.42412 (12)	0.0399	
N19	0.42952 (19)	0.70056 (13)	0.55279 (11)	0.0513	
C20	0.3356 (3)	0.61733 (19)	0.64173 (14)	0.0591	
C21	0.5486 (4)	0.8446 (2)	0.59983 (17)	0.0903	
C22	0.2647 (2)	0.50796 (14)	0.37750 (12)	0.0422	
C23	0.22417 (19)	0.45612 (13)	0.24879 (12)	0.0402	
H71	0.0500	0.3963	−0.2441	0.0557*	
H91	0.1132	−0.1320	−0.3079	0.0600*	
H101	0.2329	−0.2053	−0.1062	0.0641*	
H111	0.3097	−0.0605	0.0902	0.0601*	
H121	0.2673	0.1552	0.0875	0.0528*	
H161	0.4492	0.7442	0.1416	0.0485*	
H171	0.5265	0.8322	0.3594	0.0528*	
H201	0.3856	0.6702	0.7280	0.0885*	
H203	0.1930	0.5921	0.6121	0.0885*	
H202	0.3622	0.5286	0.6450	0.0885*	
H211	0.5837	0.8563	0.6932	0.1350*	
H213	0.4752	0.9046	0.5672	0.1350*	
H212	0.6655	0.8711	0.5708	0.1350*	
H221	0.2121	0.4466	0.4367	0.0519*	
H231	0.1484	0.3589	0.2193	0.0481*	

### Atomic displacement parameters (Å^2^)

**Table:**

	*U*^11^	*U*^22^	*U*^33^	*U*^12^	*U*^13^	*U*^23^
O1	0.0692 (6)	0.0362 (5)	0.0336 (4)	0.0168 (4)	0.0184 (4)	0.0015 (4)
C2	0.0442 (7)	0.0376 (6)	0.0332 (6)	0.0136 (5)	0.0122 (5)	0.0005 (5)
C3	0.0355 (6)	0.0348 (6)	0.0363 (6)	0.0096 (5)	0.0109 (5)	0.0019 (5)
C4	0.0397 (6)	0.0371 (6)	0.0375 (6)	0.0095 (5)	0.0095 (5)	0.0001 (5)
O5	0.0631 (6)	0.0452 (5)	0.0345 (5)	0.0183 (5)	0.0072 (4)	−0.0028 (4)
C6	0.0555 (8)	0.0498 (8)	0.0358 (6)	0.0196 (6)	0.0069 (6)	0.0016 (6)
C7	0.0571 (8)	0.0444 (7)	0.0378 (6)	0.0241 (6)	0.0105 (6)	0.0042 (5)
O8	0.1030 (10)	0.0720 (8)	0.0340 (5)	0.0396 (7)	0.0038 (5)	0.0047 (5)
C9	0.0537 (8)	0.0389 (7)	0.0534 (8)	0.0137 (6)	0.0140 (6)	−0.0039 (6)
C10	0.0543 (8)	0.0396 (7)	0.0668 (9)	0.0197 (6)	0.0150 (7)	0.0058 (6)
C11	0.0500 (8)	0.0478 (8)	0.0534 (8)	0.0194 (6)	0.0104 (6)	0.0122 (6)
C12	0.0467 (7)	0.0439 (7)	0.0382 (6)	0.0149 (6)	0.0099 (5)	0.0052 (5)
C13	0.0487 (7)	0.0361 (6)	0.0357 (6)	0.0171 (5)	0.0133 (5)	0.0045 (5)
O14	0.0936 (8)	0.0462 (6)	0.0392 (5)	0.0159 (5)	0.0266 (5)	0.0087 (4)
C15	0.0419 (6)	0.0361 (6)	0.0331 (6)	0.0161 (5)	0.0124 (5)	0.0044 (5)
C16	0.0461 (7)	0.0368 (6)	0.0379 (6)	0.0129 (5)	0.0159 (5)	0.0076 (5)
C17	0.0477 (7)	0.0367 (6)	0.0415 (7)	0.0090 (5)	0.0120 (5)	0.0012 (5)
C18	0.0423 (7)	0.0456 (7)	0.0324 (6)	0.0185 (5)	0.0077 (5)	0.0033 (5)
N19	0.0632 (8)	0.0545 (7)	0.0319 (5)	0.0193 (6)	0.0086 (5)	0.0011 (5)
C20	0.0716 (10)	0.0777 (11)	0.0347 (7)	0.0325 (9)	0.0179 (7)	0.0104 (7)
C21	0.1312 (19)	0.0643 (11)	0.0409 (9)	0.0037 (11)	0.0096 (10)	−0.0089 (8)
C22	0.0506 (7)	0.0418 (7)	0.0366 (6)	0.0160 (6)	0.0163 (5)	0.0102 (5)
C23	0.0474 (7)	0.0344 (6)	0.0385 (6)	0.0128 (5)	0.0139 (5)	0.0048 (5)

### Geometric parameters (Å, °)

**Table:**

O1—C2	1.3728 (14)	C13—C15	1.4586 (16)
O1—C13	1.3885 (15)	C15—C16	1.3937 (17)
C2—C3	1.4430 (17)	C15—C23	1.3985 (17)
C2—C7	1.3373 (18)	C16—C17	1.3762 (17)
C3—C4	1.3920 (16)	C16—H161	0.969
C3—C12	1.3958 (17)	C17—C18	1.4110 (18)
C4—O5	1.3749 (15)	C17—H171	0.944
C4—C9	1.3892 (18)	C18—N19	1.3637 (16)
O5—C6	1.3794 (17)	C18—C22	1.4121 (18)
C6—C7	1.4425 (18)	N19—C20	1.4490 (19)
C6—O8	1.2056 (16)	N19—C21	1.433 (2)
C7—H71	0.952	C20—H201	0.967
C9—C10	1.376 (2)	C20—H203	0.976
C9—H91	0.965	C20—H202	0.994
C10—C11	1.388 (2)	C21—H211	0.958
C10—H101	0.967	C21—H213	0.977
C11—C12	1.3764 (19)	C21—H212	0.956
C11—H111	0.965	C22—C23	1.3742 (17)
C12—H121	0.954	C22—H221	0.965
C13—O14	1.1970 (15)	C23—H231	0.958
			
C2—O1—C13	122.05 (10)	C13—C15—C23	123.81 (11)
O1—C2—C3	113.36 (10)	C16—C15—C23	117.98 (11)
O1—C2—C7	125.23 (12)	C15—C16—C17	121.34 (11)
C3—C2—C7	121.35 (11)	C15—C16—H161	118.6
C2—C3—C4	117.02 (11)	C17—C16—H161	120.1
C2—C3—C12	124.46 (11)	C16—C17—C18	120.99 (12)
C4—C3—C12	118.52 (12)	C16—C17—H171	118.8
C3—C4—O5	121.54 (11)	C18—C17—H171	120.2
C3—C4—C9	121.44 (12)	C17—C18—N19	121.52 (12)
O5—C4—C9	117.02 (11)	C17—C18—C22	117.38 (11)
C4—O5—C6	121.54 (10)	N19—C18—C22	121.10 (12)
O5—C6—C7	117.73 (11)	C18—N19—C20	120.85 (12)
O5—C6—O8	116.75 (12)	C18—N19—C21	120.98 (13)
C7—C6—O8	125.52 (14)	C20—N19—C21	117.41 (13)
C6—C7—C2	120.70 (12)	N19—C20—H201	109.4
C6—C7—H71	116.9	N19—C20—H203	110.3
C2—C7—H71	122.4	H201—C20—H203	109.8
C4—C9—C10	118.74 (13)	N19—C20—H202	110.5
C4—C9—H91	119.3	H201—C20—H202	109.5
C10—C9—H91	121.9	H203—C20—H202	107.3
C9—C10—C11	120.87 (13)	N19—C21—H211	108.5
C9—C10—H101	120.4	N19—C21—H213	110.0
C11—C10—H101	118.7	H211—C21—H213	109.9
C10—C11—C12	120.06 (13)	N19—C21—H212	110.5
C10—C11—H111	120.8	H211—C21—H212	109.4
C12—C11—H111	119.2	H213—C21—H212	108.5
C3—C12—C11	120.37 (12)	C18—C22—C23	120.82 (12)
C3—C12—H121	118.8	C18—C22—H221	119.6
C11—C12—H121	120.8	C23—C22—H221	119.6
O1—C13—O14	122.43 (11)	C15—C23—C22	121.45 (12)
O1—C13—C15	110.44 (10)	C15—C23—H231	118.7
O14—C13—C15	127.08 (12)	C22—C23—H231	119.9
C13—C15—C16	118.18 (11)		

### Hydrogen-bond geometry (Å, °)

**Table:**

Cg3 is the centroid of the benzoate-benzene ring (C15–C18/C22/C23).

**Table:**

*D*—H···*A*	*D*—H	H···*A*	*D*···*A*	*D*—H···*A*
C9—H91···O8^i^	0.96	2.49	3.449 (2)	171
C7—H71···Cg3^ii^	0.95	2.84	3.429 (2)	121
C20—H202···Cg3^iii^	0.99	2.91	3.777 (2)	146

Symmetry codes: (i) −*x*, −*y*, −*z*−1; (ii) −*x*, −*y*+1, −*z*; (iii) −*x*+1, −*y*+1, −*z*+1.
